# Spectral discrimination of pediatric SF188 and adult glioblastoma stem cells by deep learning–enhanced Raman profiling

**DOI:** 10.3389/fonc.2026.1748133

**Published:** 2026-02-02

**Authors:** Lennard M. Wurm, Björn Fischer, Volker Neuschmelting, Roland Goldbrunner, Roland S. Croner, Michal W. Jagielski, Dominik Laue, Wolfgang Ertel, Michael C. Hacker, Jakub Dybaś, Ulf D. Kahlert

**Affiliations:** 1 Department of Traumatology and Reconstructive Surgery, Charité - Universitätsmedizin Berlin, Corporate Member of Freie Universität Berlin, Humboldt-Universität zu Berlin and Berlin Institute of Health, Berlin, Germany; 2 Medical Faculty, Heinrich-Heine University Düsseldorf, Düsseldorf, Germany; 3 Heinrich Heine University Düsseldorf, Faculty of Mathematics and Natural Sciences, Institute of Pharmaceutics and Biopharmaceutics, Düsseldorf, Germany; 4 RSS, Raman Spectroscopic Services, Meerbusch, Germany; 5 Department of Neurosurgery, University Hospital Cologne, Cologne, Germany; 6 Department of Molecular and Experimental Surgery, Clinic of General- Visceral-, Vascular and Transplantation Surgery, University Hospital Magdeburg and Medical Faculty Otto-von-Guericke University, Magdeburg, Germany; 7 Department of Orthopedics and Trauma Surgery, Asklepios Klinik Birkenwerder, Birkenwerder, Germany; 8 Jagiellonian Center for Experimental Therapeutics, Jagiellonian University, Krakow, Poland

**Keywords:** pediatric glioblastoma, glioblastoma stem cells, molecular fingerprinting, raman spectroscopy, deep learning, artificial intelligence, label-free diagnostics, precision neuro-oncology

## Abstract

**Background:**

Pediatric and adult glioblastomas (GBM) represent biologically distinct entities requiring age-tailored therapeutic strategies. However, rapid and non-invasive methods to distinguish these molecular subtypes remain an unmet clinical need. This study evaluates the potential of confocal Raman spectroscopy combined with deep learning as a label-free diagnostic tool to differentiate pediatric from adult GBM based on intrinsic biochemical fingerprints.

**Methods:**

We acquired n=1,382 Raman spectra from a cohort of six patient-derived GBM neurosphere cell lines, comprising a pediatric model (SF188) and five adult-origin lines. A multilayer perceptron (MLP) neural network was trained to classify spectra by age group. To ensure rigorous validation and generalizability, performance was assessed on a strictly held-out external test set (20% of data), completely excluded from model optimization.

**Results:**

The deep learning model successfully differentiated pediatric from adult GBM signatures with an overall accuracy of 83.6% and an ROC AUC of 0.855 on the independent test set. Spectral analysis revealed distinct vibrational modes, highlighting significant variations in lipid, protein, and nucleic acid content between age groups. Notably, the model achieved a high sensitivity for the pediatric phenotype (91.4% identification rate) .

**Conclusion:**

This proof-of-concept study demonstrates that Raman spectroscopy, augmented by deep learning, can identify age-related molecular variations in GBM without extrinsic labeling. By capturing the unique biochemical landscape of pediatric versus adult tumors, this approach lays the foundation for rapid, automated, and objective diagnostic workflows in precision neuro-oncology.

## Introduction

Glioblastoma, a formidable and heterogeneous form of primary brain cancer, remains a critical challenge in oncology due to its complex molecular landscape and dismal prognosis (1). Rapid differentiation between pediatric and adult-type high-grade gliomas is clinically critical, as these entities require distinct adjuvant therapy protocols and exhibit significantly different prognoses. Intraoperative stratification could therefore aid in immediate surgical and therapeutic decision-making. Notably, pediatric glioblastomas are biologically distinct from adult-onset cases, exhibiting unique genetic and epigenetic alterations and clinical behavior (2). These differences have prompted the latest WHO classification to formally separate pediatric-type diffuse high-grade gliomas from adult-type glioblastomas (1). However, the molecular disparities between pediatric and adult GBM continue to be unraveled. Pediatric high-grade gliomas (pHGG) often harbor defining mutations in histone H3 genes (H3F3A, HIST1H3B), such as the H3 K27M or G34R/Vsubstitutions that reprogram the epigenome and drive aggressive, developmentally stalled tumor phenotypes (3–5). These alterations are rarely seen in adult GBMs, which more frequently exhibit aberrations like EGFR amplification, TERT promoter mutations, or PTEN loss on an IDH-wildtype background (6). Concordantly, pediatric cases can show disruptions in pathways (e.g. TP53 or PDGFRA) distinct from the adult counterpart (7, 8). Beyond genomic drivers, emerging evidence indicates fundamental metabolic and microenvironmental divergences between age groups (9). A recent integrated metabolomics study revealed that pediatric gliomas rely more on aerobic glycolysis (glucose metabolism), whereas adult GBMs are comparatively enriched in fatty acid oxidation pathways (10, 11). Likewise, while diffuse gliomas in children have historically been considered immunologically “cold” tumors with low mutational burden and fewer neoantigens, certain pediatric subtypes (e.g. diffuse midline gliomas) paradoxically display pronounced inflammatory infiltrates (12). Clinically, pediatric patients often respond better to standard treatments and achieve slightly longer survival than adults, yet they face unique long-term sequelae, whereas adult GBM remains almost uniformly fatal despite aggressive multimodal therapy (12). Taken together, these multi-faceted disparities underscore that pediatric and adult glioblastomas are distinct entities, demanding innovative approaches to decipher their specific molecular signatures and guide tailored therapies (12, 13).

Raman spectroscopy (RS) has emerged as a powerful label-free analytical technique to interrogate the biochemical composition of cells and tissues, offering a “molecular fingerprint” based on vibrational signatures of proteins, lipids, nucleic acids and other metabolites (14). Because RS can be applied *in vivo* or ex vivo without exogenous dyes or contrast agents, it is particularly attractive for brain tumor analysis where traditional biopsies or stains are challanged by sample preparation efforts and time constraints (15). Indeed, recent advances in Raman-based intraoperative tools have shown high accuracy in differentiating tumor from normal brain tissue. For example, wide-field RS imaging has been used to detect residual glioblastoma cells at surgical margins, achieving ~90% sensitivity and 95% specificity in real time (16). Likewise, RS mapping of fresh GBM specimens can delineate intratumoral heterogeneity, distinguishing viable tumor regions from necrosis and even identifying distinct metabolic subpopulations within the tumor (17). These studies highlight RS’s potential to faster capture subtle molecular differences than histopathology.

In parallel, deep learning and other machine learning algorithms have revolutionized the analysis of complex biomedical data, enabling automated pattern recognition in multi-dimensional datasets (18–20). Neural networks, in particular, excel at extracting subtle, non-linear spectral features from large Raman datasets, facilitating accurate classification of tissue states and disease subtypes (21). The synergy of RS and artificial intelligence has already demonstrated remarkable capabilities in neuro-oncology. For instance, machine-learning–assisted Raman assays have identified glioma stem-cell like states based on intracellular spectral signatures, with over 91% accuracy in distinguishing undifferentiated tumor cells (characterized by higher lipid content and altered protein structure) from more differentiated phenotypes (11). Similarly, a convolutional neural network–driven Raman analysis was recently shown to achieve high-precision diagnostic classification of GBM tissue (22). Beyond Raman, other label-free platforms are being augmented by AI to subtype gliomas (23–26), underscoring a broader trend of label-free, AI-driven diagnostic technologies rapidly advancing the molecular classification of brain tumors without the need for time-consuming histology (27).

By capturing the intrinsic chemical composition of tumor cells and leveraging neural networks to recognize complex spectral patterns, AI-driven technologies can directly probe the “fingerprints” of age-specific tumor biology (28–30). However, until recently no study had comprehensively applied Raman spectroscopic deep learning (RS-DL) specifically to differentiate pediatric vs. adult glioblastoma. The present work addresses this gap by employing Raman microspectroscopy on a panel of patient-derived pediatric and adult GBM neurosphere cell lines, combined with a deep neural network classifier, to uncover age-related molecular signatures of a widely used SF188 pediatric model. We hypothesize that this label-free spectroscopic approach will reveal distinctive spectral profiles corresponding to the known genetic, proteomic, and metabolic differences between pediatric and adult GBM. Our findings contribute to a growing body of evidence that advanced label-free optical technologies, empowered by AI, can illuminate the subtle heterogeneity of glioblastomas across age groups, paving the way for improved diagnostic precision and personalized neuro-oncologic care.

## Methods

### Cell lines and culture conditions

We utilized six patient-derived GBM cell lines to represent adult and pediatric tumors. Five lines were of adult origin (HSR-GBM1 & JHH-520, generously provided by CG Eberhart, Johns Hopkins Hospital, Baltimore, MD, USA; BTSC-407, BTSC-233 generously provided MS Carro, University Freiburg, Germany, NCH644 generously provided by C Herold-Mende, University Heidelberg, Germany), and one line (SF188) was derived from a pediatric GBM. All cells were cultured as neurospheres *in vitro* to recapitulate stem-like tumor cell characteristics. The neurosphere cultures were maintained in Dulbecco’s Modified Eagle Medium (DMEM, no pyruvate) supplemented with 30% Ham’s F-12 nutrient mix, 2% B27 supplement, 20 ng/mL human basic fibroblast growth factor (bFGF), 20 ng/mL human epidermal growth factor (EGF), 5 μg/mL heparin, and 1× antibiotic-antimycotic. Cells were grown at 37 °C in a humidified 5% CO_2_ incubator. To mimic the cellular heterogeneity of GBM, each cell line was studied both in an undifferentiated (stem-like) state and after induction of differentiation. Differentiation was triggered by treating neurospheres with 50 ng/mL recombinant BMP4 for 48 h, which promotes glial differentiation (31). All cell handling was performed in sterile conditions, and cell lines were periodically tested to ensure absence of Mycoplasma contamination.

### Confocal Raman spectroscopy acquisition

After culturing, cells were prepared for Raman microspectroscopy using a standardized protocol to ensure spectral quality. Prior to measurements, cells were washed twice with phosphate-buffered saline to remove culture medium (and phenol red) that could introduce fluorescence background. A small volume of the cell suspension (~80 μL), kept on ice, was placed onto a Raman grade calcium fluoride (CaF_2_) slide which served as a non-interfering substrate. Spectroscopic measurements were carried out on an alpha 300R confocal Raman microscope (WITec GmbH, Ulm, Germany) equipped with a Zeiss W Plan-Apochromat 63×/1.0 NA dipping objective. The excitation source was a 532 nm single-mode laser, delivered via a single-mode optical fiber to the microscope. The system included a spectrometer (WITec UHTS 300) with a 600 lines/mm diffraction grating, coupled to a thermoelectrically cooled CCD detector (Andor iDus, deep-depletion CCD at −60 °C). This configuration provided an average spectral resolution of ~3.8 cm^−1 per pixel.

For each cell, three distinct points were measured to account for intra-cellular variability, with the objective immersed directly into the droplet of cell suspension on the CaF_2_ slide. Spectra were acquired using a laser power of ~20 mW at the sample and an integration time of 20 seconds per spectrum (accumulated from ten exposures of 2 s each). These settings provided a good signal-to-noise ratio while minimizing photodamage. All spectra were collected in the “fingerprint” wavenumber range of 600–1800 cm^−1, which contains the most informative vibrational bands of cellular biomolecules. The Raman microscope was operated with WITec Five software (v5.3) for instrument control and initial data acquisition.

### Spectral preprocessing and data analysis

Following acquisition, raw Raman spectral data were preprocessed to remove artifacts and normalize the signals prior to analysis. Spectra containing obvious artifacts (e.g., cosmic ray spikes or strong fluorescence) were excluded or corrected. A baseline correction was applied to each spectrum using the rubber-band algorithm to subtract broad fluorescence backgrounds. After baseline removal, each spectrum was vector-normalized (unit norm) to account for intensity differences and focus on relative spectral features. The resulting preprocessed spectra primarily reflect the relative peak intensities of various molecular vibrations in the cells.

For spectral interpretation, we also computed a smoothed second-derivative spectrum for representative average profiles from each group. A Savitzky–Golay filter (window size 9, polynomial order 3) was applied to obtain the negative second derivative, which enhances spectral features by sharpening peaks. This helped in precisely identifying peak positions and assigning them to molecular bonds by comparison with literature values. **Table 1** summarizes the key Raman peaks that differed between pediatric and adult GBM cells in our study, along with their tentative biochemical assignments based on prior studies.

**Table 1:** Raman *sp*ectroscopic differences found in this study and the biological allocations by reference literature (biological verification was not applied in this study).

Raman peak (rel. 1/cm)	Biological allocations (32)
621	Phosphate backbone (DNA/RNA) (33)
643	Tyrosine (ring breathing) (34)
672	Phosphate backbone (DNA/RNA) (33)
719	Cholesterol (steroid ring breathing) (35)
751	Tryptophan (indole ring vibrations) (36)
781	Phenylalanine (37)
837	Proteins (Amide III, in-plane N-H bending and C-N stretching) (38)
872	Saccharides (39)
889	Glykogen (40)
936	Proline (41)
972	Phospholipids (39)
1001	Phenylalanine (34)
1031	Lipids (37)
1065	C-C Stretching in lipids (42)
1111	C-C Stretching in lipids (42)
1125	Desoxyribose (DNA/RNA) (43)
1143	Lipids (44)
1156	Proteins (Amide III) (45)
1172	Proteins (Amide III) (45)
1207	Tryptophan (45)
1259	Proteins (Amide III) (45)
1301	CH2 Twisting in lipids (46)
1314	CH2 Twisting and Wagging in lipids (42)
1326	Guanine (DNA/RNA) (43)
1338	Collagen fibers (47)
1369	CH3 Deformation in lipids (45)
1403	COO-groups (Carboxylates in amino acids) (35)
1445	CH2 Deformation in lipids and proteins (34)
1512	NADH (34)
1524	Chromophores like cytochromes (36)
1553	Proteins (Amide II) (44)
1582	Chromophores like cytochromes (28)
1618	Proteins (Amide I) (26)
1657	Proteins (Amide I, C=O Stretching) (26)
1745	Ester C=O Stretching (Triglycerides in lipids) (39)

### Deep learning classification

In total, 1382 Raman spectra were collected from the six GBM cell lines (adult and pediatric). To construct a balanced training set for classification, we randomly selected an equal number of 364 spectra from the adult group and the pediatric group. (Notably, since only one pediatric cell line was available, all of its spectra were used and a subset of adult spectra was chosen to match that count.) The balanced dataset was then split into training (80%) and testing (20%) sets using a stratified random split. To avoid bias, no spectra from a given cell were shared between training and test sets. Prior to modeling, features were standardized: each spectrum’s feature vector (intensities at each wavenumber or principal component, see below) was scaled to zero-mean and unit-variance using scikit-learn’s StandardScaler.

Because Raman spectra are high-dimensional and can be noisy, we performed dimensionality reduction using principal component analysis (PCA). The training data were transformed by PCA, retaining the top 20 principal components which captured 88.75% of variance while smoothing high-frequency noise. This reduced representation was used as input for the deep learning models. The same PCA transformation (using eigenvectors from the training set) was later applied to the test spectra.

We implemented a multi-layer perceptron (MLP) neural network for binary classification (adult vs. pediatric) using Python scikit-learn libraries. A hyperparameter search was conducted by evaluating various network architectures in cross-validation on the training set. We explored networks with 1 to 10 hidden layers, and with 2 up to 100 neurons per hidden layer, using rectified linear unit (ReLU) activation functions. The Adam optimizer was used for training with default learning rate and other parameters. Ten-fold cross-validation was employed on the training data to determine the optimal model complexity and to prevent overfitting. The best-performing architecture was an MLP with five hidden layers, each containing five neurons. This compact network was chosen for final evaluation due to its stability and superior performance (average cross-validation accuracy ~66%) relative to other tested architectures during cross-validation accuracy and simplicity. Finally, the held-out test set spectra (transformed by PCA and scaling as above) were fed into the trained MLP to obtain classification predictions, which were compared against true labels to assess performance. The test set consisted of external data points that were completely excluded from the training and cross-validation process to prevent data leakage. To ensure the technical validity and generalizability of our classification model, we implemented a rigorous data separation protocol. The dataset (spectra) was split into a training set and a strictly independent, external test set. Crucially, all preprocessing parameters, including the StandardScaler for feature normalization and the PCA for dimensionality reduction, were calculated solely based on the training data. These parameters were then applied to the held-out test set to prevent any data leakage. The final model performance was evaluated exclusively on this unseen test set, providing a robust measure of the model’s ability to discriminate the pediatric SF188 phenotype from adult glioblastoma signatures.

## Results

### Raman spectroscopic differences between pediatric and adult GBM cells

**Figure 1:**
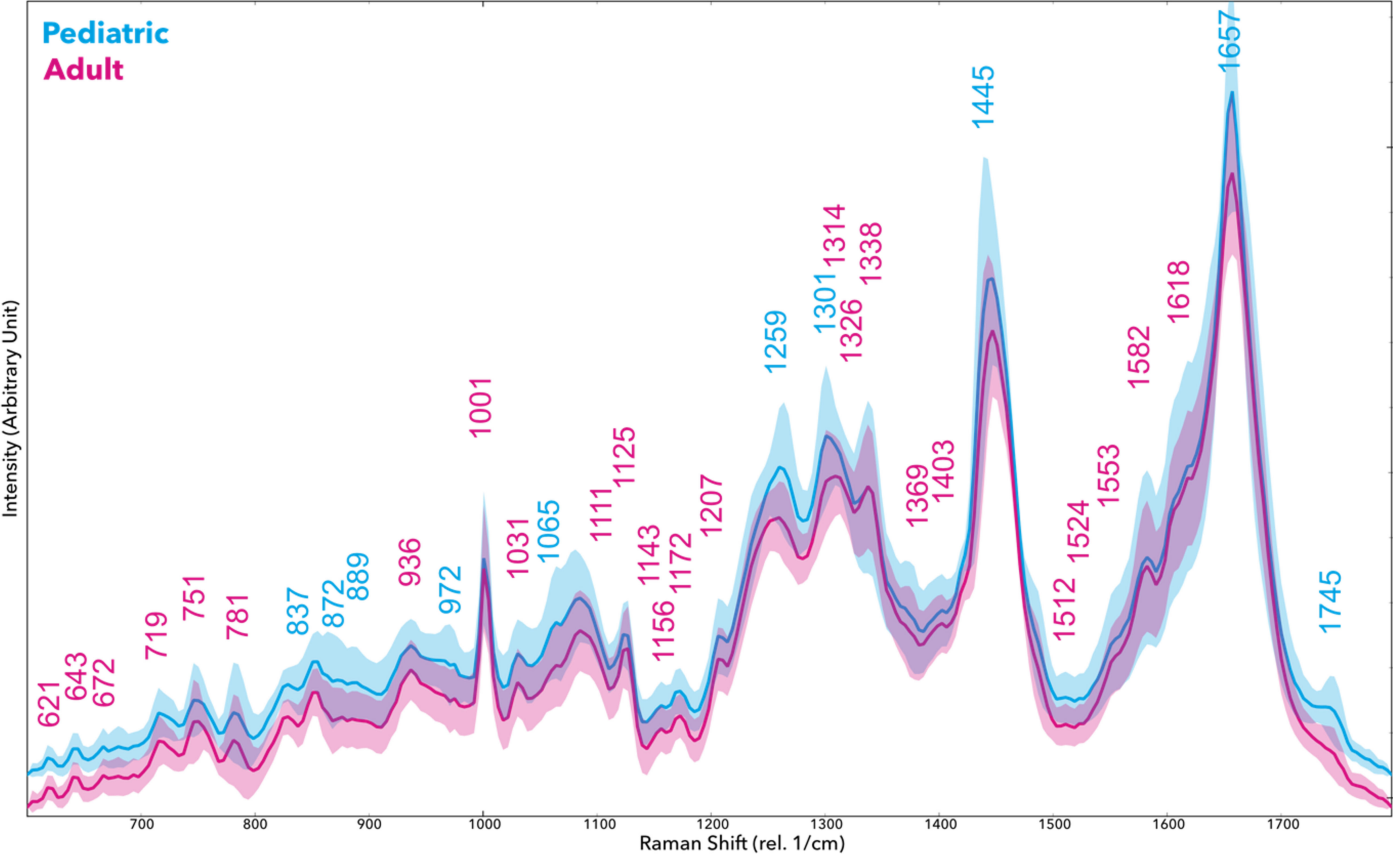
Raman *sp*ectroscopy of pediatric (blue) and adult (red) Glioblastoma cell lines (rel. 1/cm, arbitrary units). Shaded areas indicate the standard deviation. Spectra were vector-normalized; vertical alignment is shifted to prevent overlapping.

**Figure 2:**
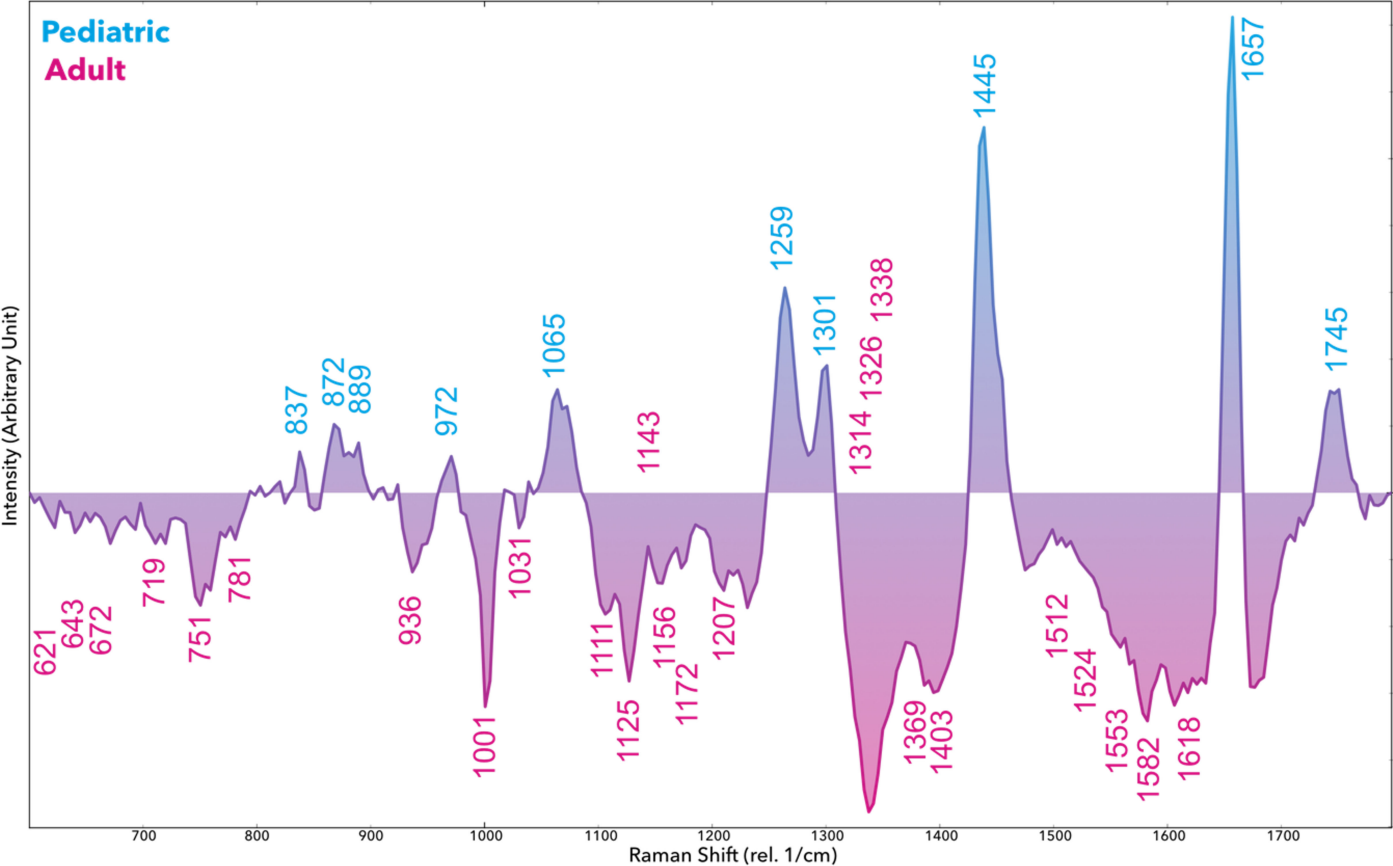
Subtraction graph shows elevated Raman *sp*ectroscopic intensities of pediatric (blue) and adult (red) Glioblastoma cell lines (rel. 1/cm, arbitrary units). Spectra were vector-normalized.

The Raman spectral profiles of pediatric vs. adult GBM cell lines revealed clear differences in several vibrational bands, indicating underlying biochemical disparities between the groups. **Figure 1** shows the representative Raman spectra averaged for the pediatric-derived SF188 line versus the aggregate of adult lines, with major peaks annotated (**Figure 1**). We observed that many spectral features were of higher intensity in the pediatric GBM cells compared to adults, especially those associated with nucleic acids, proteins, and certain lipids (**Figure 2**). For example, the pediatric spectra exhibited pronounced peaks around 621 and 672 cm^−1 (attributed to DNA/RNA phosphate backbone vibrations). Peaks in this range are commonly associated with nucleic acid content. Similarly, the pediatric line showed stronger signals at ~781–782 cm^−1 (ring breathing of DNA bases or symmetric phosphodiester vibration) and at 1125 cm^−1 (deoxyribose vibrational mode), consistent with a higher nucleic acid or nucleoprotein content in these cells. These findings suggest that the pediatric GBM cells may have increased DNA/RNA synthesis or density relative to adult cells.

**Figure 3:**
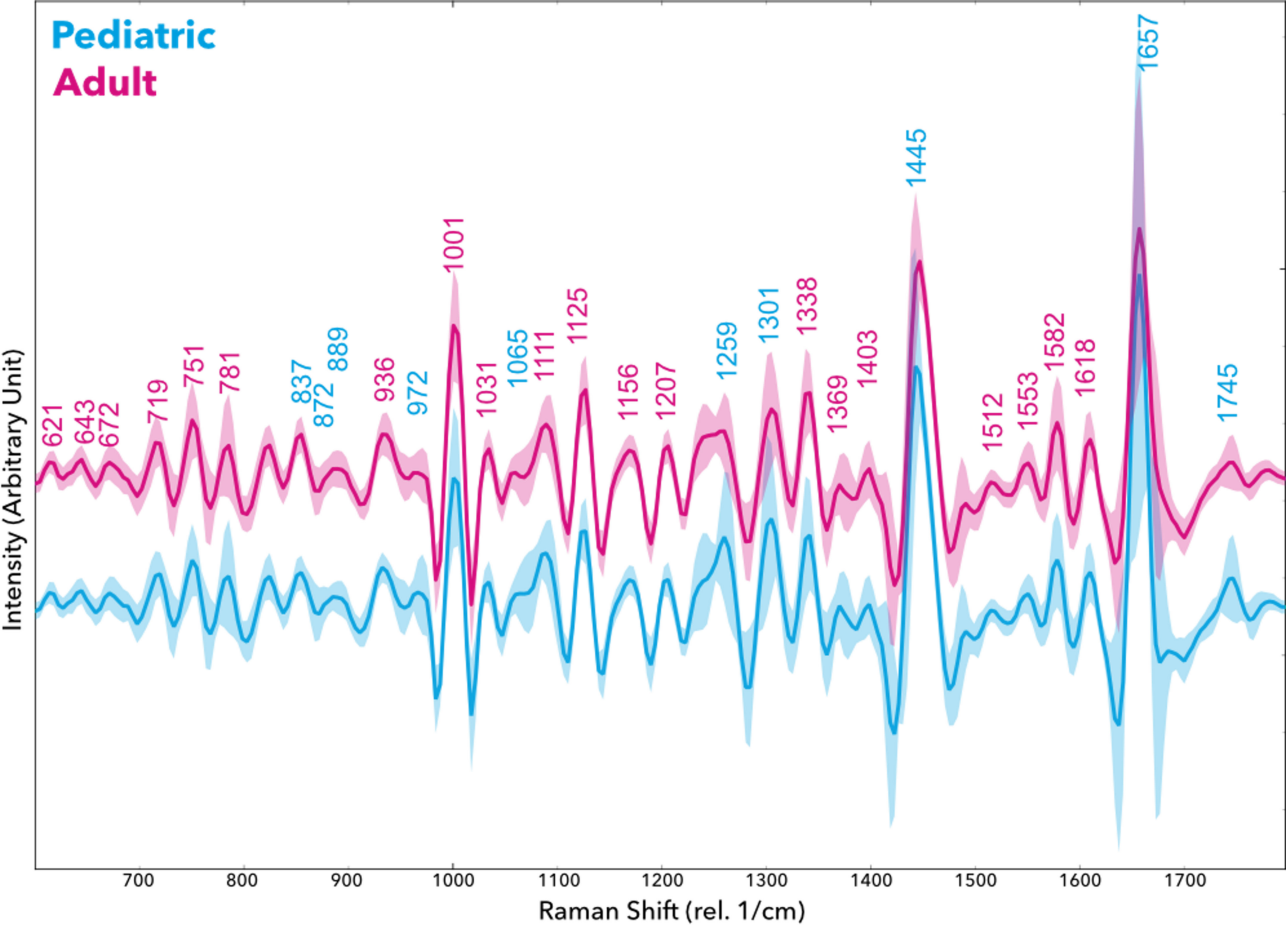
Negative second derivate filtered by Savitzky-Golay Filter to reduce noise shows peak attribution of pediatric (blue) and adult (red) Glioblastoma cells (rel. 1/cm, arbitrary units). Shaded areas indicate the standard deviation. Spectra were vector-normalized; vertical alignment is shifted to prevent overlapping.

Differences were also evident in protein-associated Raman bands (**Figure 3**). The pediatric cell spectra had higher intensity in the aromatic amino acid region (e.g., a peak near 1001 cm^−1 corresponding to phenylalanine, and a peak at 751 cm^−1 associated with tryptophan). Furthermore, amide-related peaks differed: the Amide III region (~1230–1300 cm^−1) and Amide I band (~1655 cm^−1, C=O stretching in proteins) both showed variation between the two groups. In particular, the pediatric spectra showed elevated intensity around 1657 cm^−1 (protein Amide I) relative to adults, suggesting altered protein expression or secondary structure content. Another notable difference was observed at 1445 cm^−1 (CH_2_ bending mode), a vibrational mode present in both lipids and proteins; this peak was more prominent in the pediatric line as well. An increased 1445 cm^−1 signal could indicate a higher concentration of cellular lipids or proteins with methylene-rich side chains in pediatric cells.

Lipid-associated vibrations contributed several distinguishing features. Pediatric GBM cells showed strong peaks around 1300 cm^−1 (specifically 1301 cm^−1, assigned to CH_2_ twisting in lipid chains) and 1745 cm^−1 (ester C=O stretching in lipids). These peaks imply differences in membrane lipid composition or lipid abundance. The adult GBM spectra, on the other hand, exhibited relatively higher intensities at certain bands that may relate to metabolic cofactors. For instance, peaks near 1512 cm^−1 (associated with NADH) and 1582 cm^−1 (linked to cytochrome *via* C=C stretching in porphyrin rings) were comparatively elevated in the adult group. NADH and cytochromes are involved in cellular respiration and metabolic activity, hinting that adult GBM cells might have distinct metabolic states or oxidative stress levels compared to the pediatric cells. We also noted a peak at 719 cm^−1 (cholesterol or other lipid ring vibrations) that differed between groups, and peaks in the 1330–1340 cm^−1 range (possibly collagen or other extracellular matrix components) that were more pronounced in adult-derived cell spectra. A summary of key Raman peaks differentiating the pediatric and adult samples, along with their putative biochemical assignments based on literature, is provided in **Table 1**. Overall, these spectral differences underscore that the pediatric GBM line (SF188) has a distinct molecular fingerprint, notably enriched in nucleic acids, certain amino acids, and lipids, compared to adult GBM lines.

### Deep learning model performance

We trained and evaluated an artificial neural network to classify Raman spectra as pediatric vs. adult GBM. The following performance metrics describe the model’s accuracy on the independent test set, which the model had never encountered during the training phase. The final model, a multilayer perceptron with five hidden layers of five neurons each, achieved a robust performance. On the held-out test set (with equal numbers of pediatric and adult spectra), the classifier attained an overall accuracy of 83.6%, correctly distinguishing the age group of the spectra in the majority of cases. The model’s receiver operating characteristic (ROC) curve had an area under the curve (AUC) of 0.855, indicating strong separability between the two classes. At the chosen operating point, the sensitivity for detecting pediatric spectra was 91.4%, meaning the model identified ~91% of the pediatric-derived samples correctly, while the specificity for adult spectra was 76.3% (it correctly identified ~76% of adult samples). This imbalance in classification performance reflects that the model was highly adept at recognizing the single pediatric cell line’s spectral pattern, but it also erroneously labeled about 24% of adult spectra as pediatric. The precision and recall were well-balanced (F1-score = 82.9%), underscoring that misclassifications were relatively infrequent and the model was not biased towards calling one class overwhelmingly more than the other (as seen in **Table 2**).

**Table 2:** Binary classification metrics on the two-class equally distributed test dataset by the finally used Deep Learning algorithm.

Deep learning algorithm	Predicted pediatric	Predicted adult
Actual Pediatric	91,4%	8,6%
Actual Adult	23,7%	76,3%

**Figure 4:**
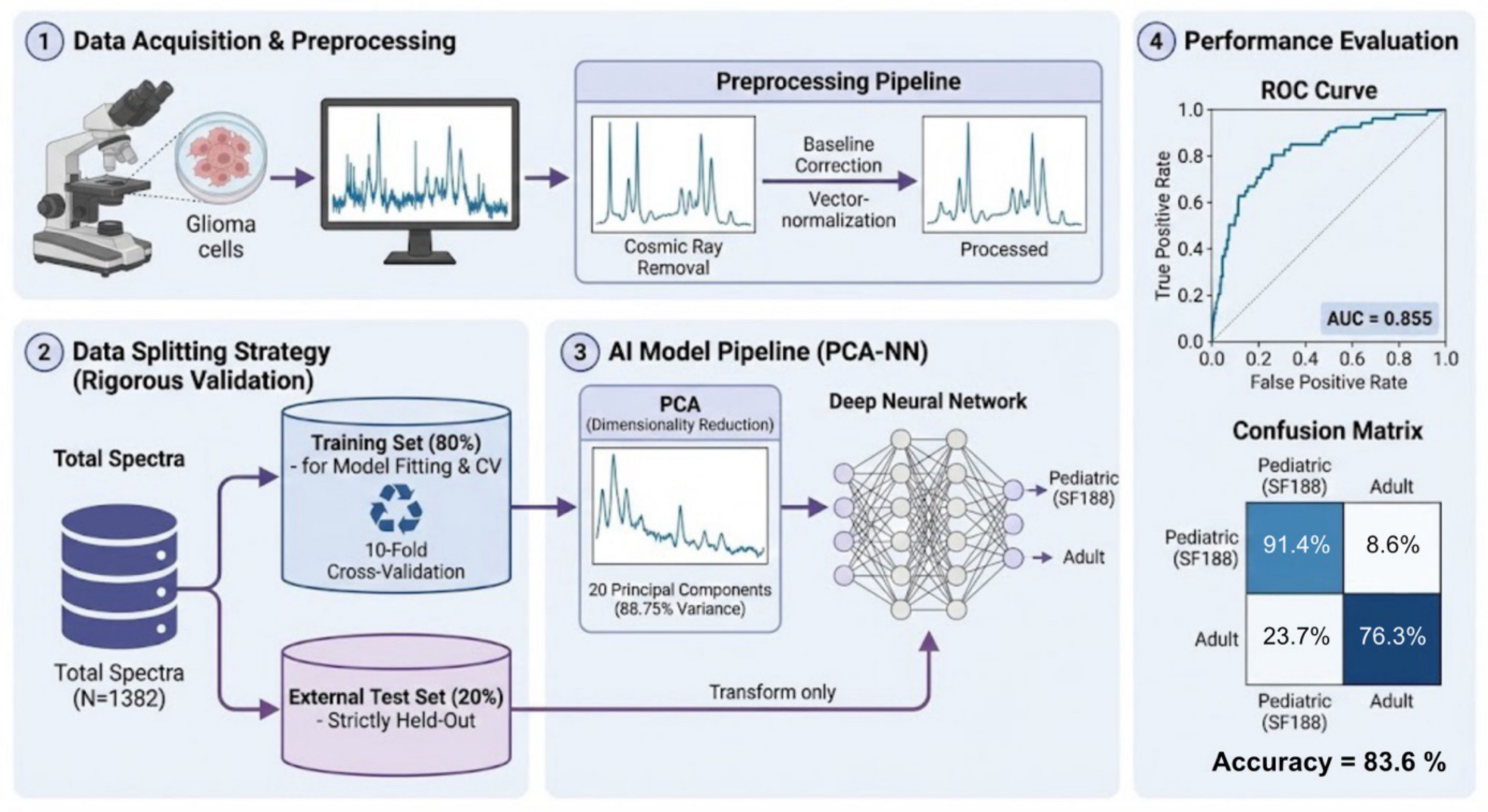
Methodology applied in this study. Confocal Raman *sp*ectroscopy can distinguish a pediatric glioblastoma subtype SF188 from five adult glioblastoma cell lines.

**Figure 4** illustrates the DL metholody applied in this study. The chosen MLP configuration (5×5 architecture) is comparatively simple, suggesting that the spectral differences captured by Raman were sufficiently clear that a complex model was not necessary for good performance. The success of this classifier confirms that there are reproducible spectral signatures allowing differentiation between the pediatric and adult GBM cell lines. Notably, even though our dataset was limited to one pediatric line, the model generalized well to held-out data from that line, implying that the spectral distinctions were consistent across different cells and measurements. These results provide an encouraging proof-of-concept that label-free spectroscopic fingerprints, analyzed with deep learning, can classify tumor samples by age origin with high accuracy.

## Discussion

In this study, we combined Raman spectroscopic analysis with deep learning to distinguish pediatric vs. adult glioblastoma cells, representing a novel integrative approach in neuro-oncology. Our findings demonstrate that GBM cells of different age origins possess measurably different molecular compositions that can be detected via their Raman spectra. To our knowledge, this work is the first to apply Raman spectroscopy with neural networks for classifying GBM by pediatric or adult origin, introducing a new dimension for tumor characterization. We acknowledge that the pediatric group is represented by the SF188 cell line. Consequently, our findings should be interpreted as a specific spectral profiling of the SF188 phenotype, a widely used model for aggressive pediatric high-grade glioma, rather than a generalized epidemiological signature for all pediatric tumors. However, the distinct spectral features identified (e.g., elevated nucleic acid and specific lipid bands) align well with the known high-proliferative and metabolically active biology of SF188 cells. The successful discrimination of this phenotype from a diverse panel of adult lines on external test data serves as a strong proof-of-concept for AI-assisted optical biopsy and highlights the potential of Raman technology to advance diagnostics in pediatric neurosurgery and neuro-oncology.

The Raman spectral differences we identified align with known biological disparities between pediatric and adult GBM, providing a biochemical context for the clinical and genetic differences reported in literature (48–52). For example, the pediatric GBM line (SF188) showed stronger DNA/RNA-associated signals (e.g., phosphate backbone vibrations) which could correlate with higher proliferative activity or distinct genomic content, consistent with frequent TP53 mutations and genomic instability observed in pediatric GBMs (2, 53, 54). In contrast, adult GBM spectra showed relatively higher intensities in regions linked to metabolic cofactors like NADH and cytochromes, which might reflect metabolic reprogramming or oxidative stress differences in adult tumors (10, 55). These observations, while based on a single pediatric model, hint at fundamental metabolic and biochemical distinctions that could underlie the divergent biology of pediatric vs. adult GBM.

The identified Raman peaks provide insight into the molecular drivers of these differences. Peaks around 751 cm^−1 (cytochromes) which may reflect metabolic reprogramming/oxidative stress differences and 1445 cm^−1 (CH_2_ groups in lipids/proteins) were distinct between the groups, suggesting differences lipid composition (56, 57). Adult GBM cells, for instance, may exhibit altered redox-sensitive metabolsim or membrane lipid profiles compared to pediatric cells. Such differences could influence tumor behavior, for example, lipid raft composition can affect signaling pathways and cell invasiveness. Similarly, the pediatric line’s elevated amide and nucleic acid peaks (e.g., 837 cm^−1 Amide III, 1657 cm^−1 Amide I, 1125 cm^−1 deoxyribose) indicate a distinctive proteomic and genomic state (58, 59). One intriguing possibility is that the higher nucleic acid signal in pediatric cells could relate to their epigenetic differences (such as histone mutations or DNA methylation patterns) and higher rates of DNA replication or repair activity. The lipid-associated peak at 1301 cm^−1 (CH_2_ twist) also differed, implying variations in lipid saturation or membrane order between pediatric and adult tumor cells (58, 60, 61). Such biophysical differences in cell membranes might impact how cells interact with their microenvironment or respond to therapy.

While we have tentatively linked certain spectral features to known molecular differences (for example, DNA peaks to TP53-associated genomic instability in pediatric GBM, or lipid/protein peaks to PTEN/EGFR-driven changes in adult GBM), these correlations are speculative and based on literature rather than direct evidence. It is important to emphasize that Raman peaks are broad indicators of biochemical content and cannot pinpoint specific genetic alterations. Nevertheless, the consistency of our spectroscopic findings with expected biological differences is encouraging. It suggests that Raman spectroscopy is sensitive to the integrated molecular phenotype of the cells, capturing changes in nucleic acids, proteins, and metabolites that arise from the distinct oncogenic pathways active in pediatric vs. adult GBM. Our results extend the list of evidences that pediatric brain tumor cells can be identified using Raman approaches incl. spectroscopy (29, 30, 62), but- to ouf knowledge- for the first time perfomring a comparison of stem cell populations of adult vs pediatric GBM.

Our deep learning results further underscore the feasibility of this approach. The neural network achieved a high accuracy (83.6%) in classifying spectra, validating that the spectroscopic differences are systematic and learnable by an algorithm. Notably, the model’s excellent sensitivity (91% for pediatric class) indicates that the single pediatric cell line had a highly distinct spectral profile that the network captured robustly. The lower specificity for adult class (76%) suggests a greater heterogeneity among the adult lines, some adult spectra were misclassified as pediatric, which could mean that a subset of adult cells exhibited Raman features somewhat similar to the pediatric line. This is not entirely surprising given the molecular diversity among adult GBM lines. Importantly, our use of cross-validation and an independent test set provides some assurance that the model did not simply overfit peculiarities of the dataset, but rather learned generalizable features. The relatively simple network architecture that sufficed for high performance is a positive sign for potential clinical translation, as simpler models are less prone to overfitting and easier to interpret. Additionally, the success of the classifier demonstrates the advantage of pairing modern computational techniques with spectroscopy: the neural network can discern subtle, multi-variable patterns in the Raman data that may be difficult to detect by eye or by conventional analysis. This highlights an important point for future work, advanced algorithms can enhance the diagnostic power of optical methods in oncology.

Despite the promising results, our study has several important limitations. First, the experimental design involved only one pediatric GBM cell line versus five adult lines. This severe imbalance in sample representation means that our findings for “pediatric” GBM are essentially based on a single biological sample (SF188). While we took multiple measurements and the model was validated on held-out spectra, it is possible that some observed spectral differences are specific to SF188 and not generalizable to all pediatric GBMs. The classifier might be learning the signature of this particular cell line (or its tissue of origin) rather than a universal pediatric GBM signature. Thus, caution is warranted in extrapolating our results to the broader population of pediatric tumors. Expanding the study to include additional pediatric GBM cell lines (and adult lines) will be critical to verify that the discriminative features hold across diverse genetic backgrounds.

Second, although conducted on 3D *in vitro* models know to recapitulate stem cell population and tumor tissue more physiological than classical monolayer cultures, our work was conducted on cell line models rather than primary cultures. Moreover, we utilized the stem cell culture technology to purify tumor cell population, and do not recapitulate tumor microenvirnmental paramters *in vivo* such as fluidics, biophysics or interantion with other cell types of the tumor. These lack of important factors influence the cellular metabolism and composition, and therefore the Raman spectra. The *in vitro* setting allowed us to measure clean cell suspensions on CaF_2_ substrates, which is ideal for spectral quality but different from how spectra would be acquired in a surgical or clinical context (e.g., on fresh tissue *in situ* or *in vivo*). As such, our accuracy of 83.6% on cell lines is an optimistic scenario; performance on real patient samples could differ.

Furthermore, although we attempted to minimize overfitting through cross-validation, the total number of independent biological samples (i.e. cell lines) was small. Deep learning models can inadvertently capture batch effects or lab-specific artifacts (63). A more rigorous validation would involve an independent test on completely new cell lines. Additionally, our current model treats the classification as a binary problem (pediatric vs adult); however, real-world use might require distinguishing multiple categories (including low-grade vs high-grade or other subtypes) and coping with intra-tumor heterogeneity.

Lastly, we acknowledge that our spectral assignments and biological interpretations (linking peaks to specific molecules or pathways) are tentative. The Raman bands we discuss (e.g., DNA, protein, lipid peaks) are broad indicators and not unique to a single molecule. While literature was used to guide assignments, definitive identification of what causes each spectral difference would require complementary biochemical assays. We present these interpretations as hypotheses to be tested in targeted experiments (for instance, by correlating Raman data with proteomic or metabolomic profiling) (64). The current study should be viewed as an initial demonstration of feasibility and exemplify the potential of Raman for modularly tailoring the future clinical care of young brain tumor patients.

## Conclusion

We have shown that label-free Raman spectroscopic profiles, combined with deep learning analysis, can distinguish stem cells between pediatric and adult glioblastoma cells, reflecting underlying molecular differences. The pediatric-derived GBM cells in our study exhibited distinctive Raman signatures, with heightened peaks corresponding to nucleic acids, proteins, and certain lipids, whereas adult GBM cells showed unique spectral features suggestive of different metabolic and biochemical states. Our optimized neural network classifier was able to leverage these spectral differences to achieve over 83% accuracy in predicting the age origin of GBM cell line samples. The results of a one-to-one comparison of 3D grown GBM cells from adult and pediatric donors are significant as a proof-of-concept that advanced optical diagnostics augment histopathological and molecular methods in distinguishing age-related tumor subtypes. The ability to rapidly identify whether a glioblastoma is more “pediatric-like” or “adult-like” could have implications for personalized therapy, given the divergent genetic pathways and treatment responses between pediatric and adult GBMs.We emphasize that this is an early exploratory study and that further research is required to validate and extend the results.

## Data Availability

The raw data supporting the conclusions of this article will be made available by the authors, without undue reservation.
